# 

**Published:** 1898-11-05

**Authors:** 


					xjjiu. ? ine stanaara at highest purity."? Lancet. Nov. 5th, 1898.'
CADBURY'S P CADBURY'S
COOOA Jnft My COCOA
ABSOLUTELY PURE. NO DRUQS OR ALKALIES USED.
);
WWKH HOSPi.TAJL
tto. 632. Vol. XXV. LfSpTpen] Saturday,
Nov. 5th, 1898.
[SutecriptionTenru,,] palCE TWOPENCE.

				

